# Down-regulation of lncRNA CASC2 promotes cell proliferation and metastasis of bladder cancer by activation of the Wnt/β-catenin signaling pathway

**DOI:** 10.18632/oncotarget.15210

**Published:** 2017-02-09

**Authors:** Zhijun Pei, Xian Du, Yafeng Song, Lin Fan, Fuyan Li, Yan Gao, Ruimin Wu, Yijia Chen, Wei Li, Hong Zhou, Yi Yang, Jing Zeng

**Affiliations:** ^1^ Department of PET Center & Institute of Anesthesiology and Pain, Taihe Hospital, Hubei University of Medicine, Shiyan, Hubei Province, 442000, China; ^2^ Department of General Surgery II, Taihe Hospital, Hubei University of Medicine, Shiyan, Hubei Province, 442000, China; ^3^ Department of Ophthalmology, Taihe Hospital, Hubei University of Medicine, Shiyan, Hubei Province, 442000, China; ^4^ Department of Infection Control, Taihe Hospital, Hubei University of Medicine, Shiyan, Hubei Province, 442000, China

**Keywords:** bladder cancer, CASC2, Wnt/β-catenin, proliferation, metastasis

## Abstract

Long noncoding RNAs cancer susceptibility candidate 2 (CASC2) have been demonstrated as playing crucial regulatory roles in a few of cancers. However, the biological function of lncRNA CASC2 in bladder cancer are still unclear. In this study, we found that lncRNA CASC2 was significantly down-regulated in bladder cancer tissues and cell lines by quantitative real time-PCR and associated with advanced TNM stage (III/IV). Moreover, overexpression of lncRNA CASC2 remarkably reduced the cell growth, migration and invasion, as well as promoted early apoptosis of bladder cancer cell *in vitro*. Furthermore, we illustrated that lncRNA CASC2 inhibited Wnt/β-catenin signal pathway activity by decrasing the β-catenin expression and reversing the downstream target gene expression of Wnt signaling pathway. Taken together, lncRNA CASC2 plays an pivotal role in bladder tumorigenesis and progression and may act as a potential biomarker for the treatment of bladder cancer.

## INTRODUCTION

Bladder cancer is one of the most prevalent urologic malignancy and the fourth more commonly diagnosed cancer among men worldwide [1]. The patients suffering from bladder cancer are surgically treated with radical cystectomy (RC), radiation therapy, and postoperative instillation of chemotherapy or immunotherapy [2, 3]. Although there have been great improvements for treatment of bladder cancer, its great recurrence rate and extremely unsatisfactory prognosis with 5-year overall survival rates [4]. Thus, it is urgent need to perform molecular mechanisms research to discover novel molecular biomarkers for the treament of bladder cancer.

Long non-coding RNAs (lncRNAs) with a length of >200 nucleotides are highly conservative across mammalian species [5]. Recent studies have showed that lncRNAs were tightly associated in carcinogenesis and can be used as the potential biomarkers of cancer [6]. LncRNAs, such as GAS5, UCA1, NEAT1, PANDAR, HOTAIR and H19 have been demonstrated that they showed crucial roles in the progression, metastasis, recurrence and prognosis of bladder cancer [7–12]. However, the functions of lncRNAs in bladder cancer remains largely unclear.

LncRNA Cancer Susceptibility Candidate 2 (CASC2), a novel human lncRNA mapping to 10q26 in humans, has been originally characterized as a downregulated gene and acted as a tumor suppressor gene in endometrial cancer [13]. Wang et al demonstrated that CASC2 plays a tumor suppressive role in glioma via negative regulation of miR-21 [14]. In addition, low expression of long noncoding RNA CASC2 indicates a poor prognosis and regulates cell proliferation in non-small cell lung cancer [15] and renal cell carcinoma cells [16]. Besides, the long noncoding RNA CASC2 functions as a competing endogenous RNA by sponging miR-18a in colorectal cancer [17]. Meantime, long non-coding RNA CASC2 suppresses the proliferation of gastric cancer cells by regulating the MAPK signaling pathway [18]. However, the role of lncRNA CASC2 in the progression of bladder cancer is unknown and needed to be further explored.

Our results showed that lncRNA CASC2 expression levels were lower in tumor tissues than those in adjacent normal tissues and down-expressed in the bladder cancer cell lines. And overexpression of lncRNA CASC2 remarkably inhibited the growth, arrested migration and invasion, as well as induced early apoptosis and attenuated the activation of Wnt/β-catenin signal pathway in T24 and 5637 bladder cancer cells.

## RESULTS

### Expression of CASC2 was down-regulated in bladder cancer and cells

**Figure 1:**
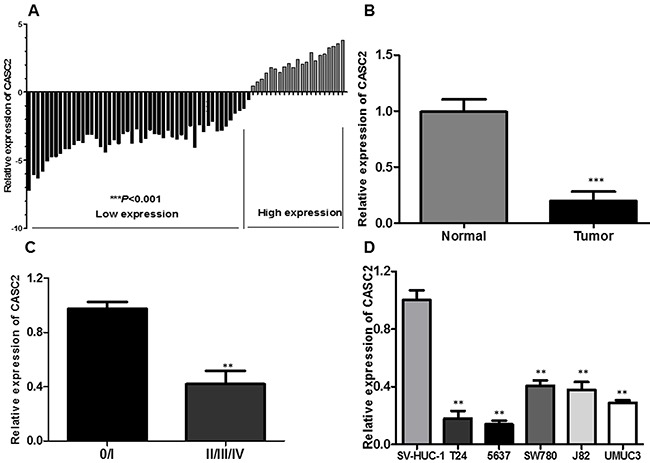
LncRNA CASC2 expression is decreased in bladder cancer **A**. Relative expression of CASC2 in 72 pairs of bladder cancer tissues and adjacent non-tumor tissues by qRT-PCR analysis. ***P<0.001 compared with non-tumor control. **B**. CASC2 expression levels were dramatically lower in patients with higher TNM stage. **C**. The expression levels of CASC2 in a panel of bladder cancer cell lines were determined by qRT-PCR and compared with that in normal urothelia cells (SV-HUC-1). **P < 0.01 compared with the SV-HUC-1 cell. **D**. qPCR analysis of CASC2 expression levels following the treatment of T24 and 5637 cells with pcDNA-CASC2. Data represent the mean ± SD from three independent experiments. *P < 0.05; **P < 0.01.

We firstly explored the relative expression level of CASC2 in bladder cancer tissues (n=72) compared with corresponding non-tumor tissues (n=72) by qRT-PCR, and normalized to GAPDH. As shown in Figure 1A, the CASC2 level was significantly decreased in bladder cancer tissues compared with corresponding adjacent non-tumorous tissues (P<0.001, Figure 1A and 1B). Moreover, down-regulated CASC2 expression was significantly correlated with advanced TNM stage (Figure 1C, P<0.01). Then, we examined the expression of CASC2 in a panel of bladder cancer cells (T24, 5637, SW780, J82 and UMUC3) and normal urothelial cells (SV-HUC-1) by qRT-PCR. As shown in Figure 1C, the result of qRT-PCR exhibited that the bladder cancer cells showed low expression of CASC2 compared with the normal urothelial cells (P<0.01, Figure 1D). These data suggested that abnormal CASC2 expression may be associated with bladder cancer pathogenesis.

### Overexpression of CASC2 inhibits proliferation of T24 and 5637 cells *in vitro*

**Figure 2:**
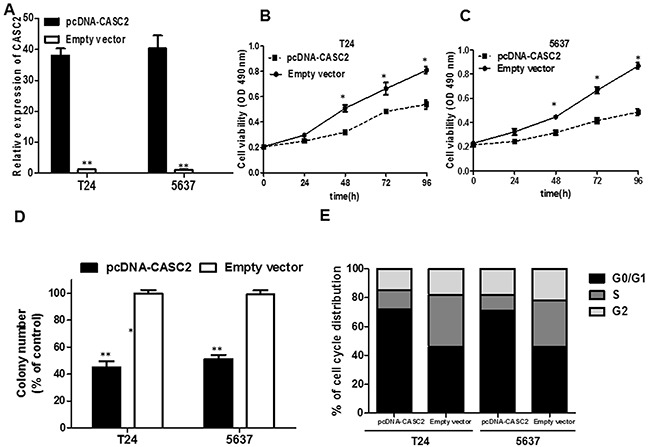
The effect of CASC2 overexpression on cell viability and cell cycle **A**. The pcDNA-CASC2 significantly up-regulated the expression level of CASC2 in T24 and 5637 cells. **B**. MTS assay showed overexpression of CASC2 inhibited cell proliferation of T24 and 5637 cells. **C**. Colony formation assay measuring colony formation of CASC2 overexpression cells. Colony number was normalized to that obtained with cells transfected with empty vector, which was set to 100%. Overexpression of CASC2 significantly decreased the colony-forming ability of T24 and 5637 cells. **D**. Effect of CASC2 overexpression on the T24 and 5637 cell cycle. Cell cycle distribution was measured by propidium iodide staining followed by flow cytometry. The two cell had cell-cycle arrest at the G0-G1 phase compared with cells transfected with Empty vector. Each assay was performed in triplicate. Data are mean ± SD. * P<0.05, **P<0.01.

We detected that lncRNA CASC2 expression was comparatively lower in T24 and 5637 cell lines than that in SW780, J82 and UMUC3 cancer cell lines (Figure 1C). Therefore, we selected T24 and 5637 cell lines for the following biological function studies. To further explore the role of CASC2 in bladder cancer cells, the lncRNA pcDNA-CASC2 was designed and transfected into T24 and 5637 cells. As shown in Figure 2A, cells transfected with pcDNA-CASC2 presented a remarkably increased mRNA expression level of CASC2 compared with the empty vector group in both cells by qRT-PCR (P<0.05; Figure 2A). To determine the effect of CASC2 on the proliferation of bladder cancer cell *in vitro*, MTS assays showed that the overexpression of CASC2 apparently abrogated the proliferation rate of T24 and 5637 cells (P<0.05; Figure 2B and 2C). Moreover, we used a colony formation assay to further examine the role of CASC2 on growth of T24 and 5637 cells. Consistently, the results showed that bladder cancer cells transfected with pcDNA-CASC2 significantly reduced the colony numbers (P<0.01; Figure 2D). Besides, flow cytometry was used following transfection to assess cell cycle distribution. The data demonstrated that the cell population in the G0/G1 phase was expanded but the S phase population was limited after the overexpression of CASC2 compared with the negative group cells (Figure 2E), further indicating that overexpression of CASC2 may restrain cancer cell growth by regulating the cell cycle. Altogether, these results clarified that CASC2 may function as a suppressor gene promoting bladder cancer cell proliferation.

### Overexpression of CASC2 increases apoptosis of bladder cancer cells

**Figure 3:**
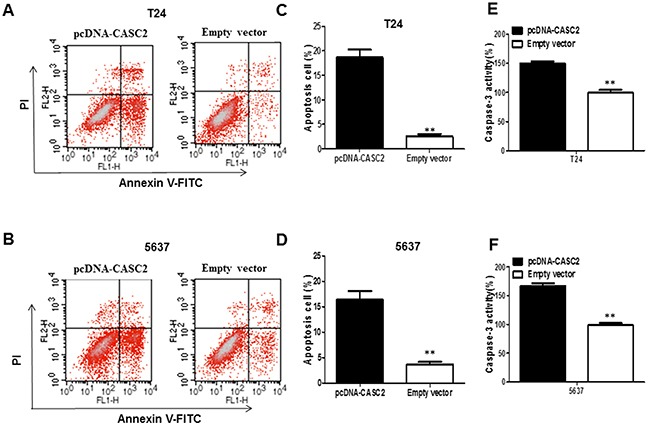
The overexpression of lncRNA CASC2 augmented the apoptosis of bladder cancer cells **A** and **B**. Apoptosis of T24 and 5637 cell lines was determined by flow cytometry. **C** and **D**. Histogram of percentage of apoptotic cells, according to (A) and (B). **E** and **F**. Caspase-3 colorimetric assay was used to detect the Caspase-3 activity; GAPDH was used as control. Each assay was performed in triplicate. Data are mean ± SD. *P<0.05, **P<0.01.

To investigate whether overexpression of CASC2 induce cell apoptosis, flow cytometry was used to analyze the cell apoptosis of bladder cancer cells transfected with pcDNA-CASC2. The results indicated that overexpression of CASC2 dramatically induced apoptosis of T24 (Figure 3A) and 5637 cells (Figure 3B), especially early apoptosis. Compared with the cells transfected with empty vector, cell apoptosis increased approximately 19.56% in T24 cells (Figure 3C), and 16.72% in 5637 cells (Figure 3D) when treated with pcDNA-CASC2. Next, we examined caspase-3 activity in bladder cancer cells in response to CASC2 overexpression. Caspase-3 is recognized to stimulate apoptosis, as one member of caspase family which are cytosolic aspartate-specific cysteine proteases involved in the initiation and execution of apoptosis. Caspase-3 colorimetric assay analysis showed that CASC2 overexpression enhanced the cleaved caspase-3 expression in T24 (Figure 3E) and 5637 cells (Figure 3F).

### Overexpression of CASC2 suppressed migration and invasion of bladder cancer cells

**Figure 4:**
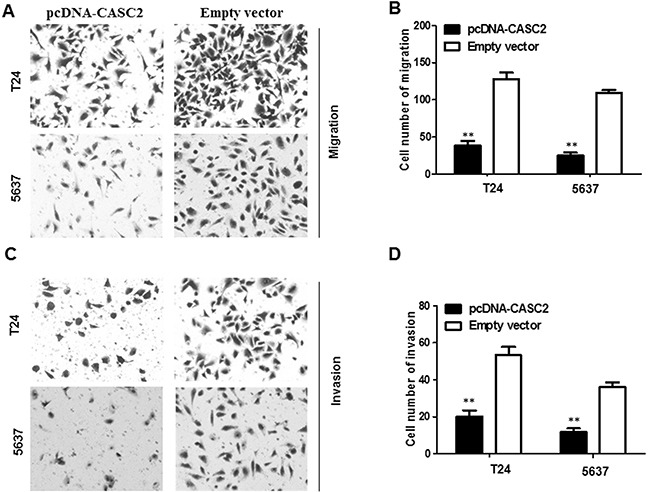
Overexpressed CASC2 reduced migration and invasion of bladder cancer cells Cell migration and invasion was determined by transwell assay. **A** and **B**. inhibition of migration of T24 and 5637 cells by overexpression of CASC2. **C** and **D**. inhibition of invasion of T24 and 5637 cells by overexpression of CASC2. Data are shown as mean ± SD. The experiments were all repeated at least three times. ** P< 0.01 compared with empty vector group.

To further probe the influence of CASC2 on bladder cancer cell migration and invasion, we conducted transwell assays in T24 and 5637 cells. Overexpression of CASC2 obviously impaired T24 and 5637 cells migration ability compared with the empty vector group (P<0.01, Figure 4A and 4B). Next, transwell invasion assay was carried out to document the effect of CASC2 on the invasiveness of bladder cancer cells. The results illuminated that overexpressio of CASC2 induced prominent suppression in ability of cell invasion compared with the empty vector group (P<0.01, Figure 4C and 4D).

### Overexpression of CASC2 inhibits Wnt/β-catenin signaling activation in bladder cancer

**Figure 5:**
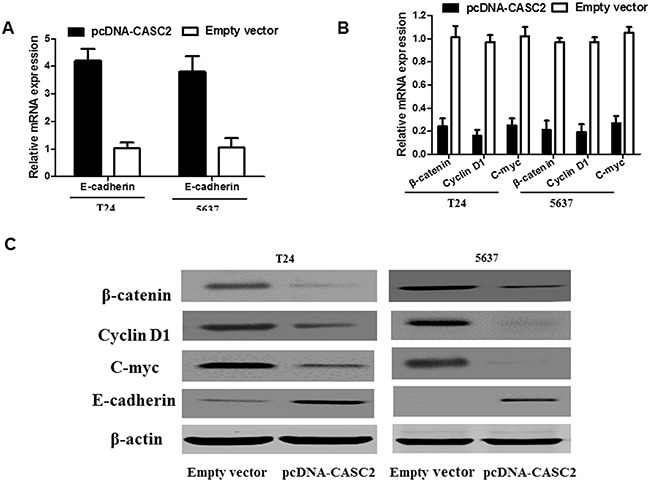
Effect of CASC2 on Wnt/β-catenin signaling pathway in bladder cancer cells T24 and 5637 cells were transfected with pcDNA-CASC2 and Empty vector for 48 h. Overexpression of CASC2 affected the Wnt/β-catenin signaling pathway. Western blot and qRT-PCR analysis showed that overexpression of CASC2 inhibited the β-catenin, cyclin D1 and c-myc expression in the T24 and 5637 cell. Each assay was performed in triplicate. Data are mean ± SD. *P<0.05, **P<0.01.

As is known, the Wnt/β-catenin signaling pathway plays a crucial role in the regulation of cell growth and migration. Therefore, using Western blot assay and qRT-PCR, we detected the effect of CASC2 on β-catenin expression and a few of the downstream genes of the Wnt/β-catenin signaling pathway, such as, cyclin D1, c-myc and E-cadherin in bladder cancer cells. The results demonstrated that the expression of β-catenin, c-myc and cyclin D1 was significantly reduced while E-cadherin expression was obviously augmented when CASC2 was overexpressed in T24 and 5637 cells (Figure 5A, 5B and 5C). Thus, these data indicated that the CASC2 could attenuate the activation level of the Wnt/β-catenin signaling pathway. These results further confirm that the downregulation of CASC2 has an effect on the biological behavior of bladder cancer cells by regulating Wnt/β-catenin signaling pathway.

## DISCUSSION

Mounting evidence exhibits that lncRNAs play more and more vital roles in a wide range of biological processes. Experimental studies have demonstrated that some lncRNAs, which may act as oncogenes or cancer suppressors, contributes to human cancer pathogenesis and progression [6]. For instance, knockdown of long noncoding RNA CCAT2 inhibits cellular proliferation, invasion, as well as induces early apoptosis by regulating EMT in glioma cells [19]. Long non-coding RNA BANCR promotes endometrial cancer cell proliferation and invasion by regulating MMP2 and MMP1 via ERK/MAPK signaling pathway [20]. These reports suggested that lncRNAs play significant roles during cancer progression.

CASC2, a recently found lncRNA, has been proved to be linked with the prognosis of cancer patients and regulate cell growth in NSCLC, glioma, colorectal cancer, gastric cancer, endometrial cancer and renal cell carcinoma cells. However, the role of CASC2 in bladder cancer is still unclear. In the present study, we manifested that lncRNA CASC2 expression was reduced in both bladder cancer tissues and cell lines and and negatively correlated with advanced TNM stage. Besides, we investigated the function of CASC2 in bladder cancer cells by applying gain-of-function approaches. MTS assay and colony formation assay showed that overexpression of CASC2 inhibited bladder cancer cell proliferation *in vitro*. Flow cytometry showed that overexpression of CASC2 could arrest the cell cycle at G1-S checkpoint and induce cell apoptosis *in vitro*. Additionally, transwell assay showed that overexpression of CASC2 suppressed bladder cancer migration and invasion. Our data is the same to all relevant studies in other cancer. Therefore, those results illuminated that lncRNA CASC2 may serve as a tumor suppressor in bladder cancer progression.

Wnt/β-catenin signaling pathway regulates various biological events in cells, such as gene expression, cell growth, metabolism, apoptosis and metastasis. Recent studies demonstrate that lncRNA could regulate tumor progression via Wnt/β-catenin signaling pathway [21]. For example, lncRNA T-UCR as a potential downstream driver of the Wnt/β-catenin pathway in hepatobiliary carcinogenesis [22]. To shed light on the precise mechanism underlying in CASC2-inhibited bladder cancer cell growth and migration, the effects of CASC2 on Wnt/β-catenin signaling pathway were explored. Our data suggested that overexpression of CASC2 inhibited β-catenin expression and reversed the activation level of the Wnt/β-catenin signaling pathway. Moreover, in terms of protein expression level and mRNA level, our results indicated that CASC2 can also inhibit several downstream target genes expression of the WNT signaling pathway, such as, cyclin D1, c-myc and E-cadherin. Based on our results, we considered CASC2 may be associated with the Wnt/β-catenin signaling pathway. Hence, our results provide a new hint for apprehending the pathogenesis of bladder cancer and a novel method for the diagnosis and treatment of bladder cancer.

In summary, our findings indicated that CASC2 was strikingly downregulated in bladder cancer tissues. Overexpression of CACS2 could inhibit bladder cancer cell proliferation, induce cell apoptosis and suppress cell migration and invasion *in vitro*. Moreover, Overexpression of CACS2 contributed to the inactivation of Wnt/β-catenin signaling pathway. Therefore, CACS2 might serve as a tumor suppressor lncRNA that abrogates growth of bladder cancer cells and inactivates the Wnt/β-catenin signaling pathway.

## MATERIALS AND METHODS

### Human tissue samples

72 pairs of bladder cancer tissues and the pair-matched adjacent normal tissues were obtained from the Taihe Hospital of Hubei University of Medicine during Jun 2014 to Jul 2015. All the patients received partial or radical cystectomy. All specimens were frozen immediately in liquid nitrogen and stored at −80 °C until RNA extraction. None of the patients from whom the samples were obtained had undergone preoperative chemotherapy or radiotherapy. Informed consents were obtained from all patients and this study was approved by the Clinical Research Ethics Committee at the Taihe Hospital of Hubei University of Medicine.

### Cell culture

Normal urothelial cells (SV-HUC-1) cells were purchased from ATCC (American Type Culture Collection). T24, 5637, SW780, J82 and UMUC3 cells were purchased from the Chinese Academy of Sciences (Shanghai, China). SV-HUC-1 cells were maintained in F12K medium (Invitrogen, Carlsbad, CA, USA) supplemented with 1% antibiotics (100 units/mL penicillin and100 μg/ mL streptomycin sulfates) and 10% fetal bovine serum (FBS; Hyclone, Logan, UT, USA). The 5637 cells and SW780 cells were cultured in RPMI-1640 Medium (Invitrogen) plus 1% antibiotics and 10 % fetal bovine serum. The UMUC3 and T24 cells were cultured in DMEM (Invitrogen) plus 1% antibiotics and 10 % fetal bovine serum. All cell lines were cultured at 37°C with an humidified atmosphere of 5% CO2 in incubator. All cell lines have been tested and authenticated by DNA (short tandem repeat genotyping) profiling before use.

### Quantitative real time RT-PCR (qRT-PCR) analysis

The total RNA was extracted from tissues or cultured cells with Trizol reagents (Invitrogen, Carlsbad, CA) according to the manufacture's guide. The total RNA was reverse transcribed into first-strand cDNA using the SuperScript III^®^ (Invitrogen) according to the manufacture's guide. Q-PCR was performed on the SYBR Premix ExTaq kit (TaKaRa) on ABIPRISM 7000 Fluorescent Quantitative PCR System (Applied Biosystems, FosterCity, CA, USA) according to the instructions. Results were normalized to GAPDH expression levels. The PCR primers were as follows: CASC2 primers forward: 5’-GCACATTGGACGGTGTTTCC-3’, reverse:5’- CCC AGTCCTTCACAGGTCAC-3’; E-cadherin primers forward: 5’-TGTAGTTACGTATTTATTTTTAGTGGCG TC-3’, reverse:5’-CGAATACGATCGAATCGAACCG-3’; Cycle D1 primers forward:5’-GAGACCATCCCCCTG ACGGC-3’, reverse: 5’-TCTTCCTCCTCCTCGGCGG C-3’; β-catenin primers forward: 5’-TGCAGTTCG CCTTCACTATG-3’, reverse: 5’-ACTAGTCGTGGAATG GCACC-3’; C-myc primers forward: 5’-GCCCAGTGAG GATATCTGGA-3’, reverse: 5’-ATCGCAGATGAAGC TCTGGT-3’; GAPDH primers forward: 5’-CGCTCTC TGCTCCTCCTGTTC-3’, reverse: 5’-ATCCGTTGACTC CGACCTTCAC-3’. All primers were obtained from Invitrogen, Shanghai, China. All qRT-PCR reactions were performed in triplicate. Relative quantification of tested gene expression was calculated and normalized by the 2^−ΔΔCt^ method.

### Transfection of cell lines

The CASC2 sequence was synthesized according to the full-length CASC2 sequence and then subcloned into a pcDNA3.1 vector (GenePharma, Suzhou, China). The empty pcDNA3.1 vector was used as the negative control. The pcDNA-CASC2 or empty pcDNA3.1 vector was transfected into T24 and 5637 cells cultured in six-well plates using by X-treme GENEHP DNA transfection reagent (Roche, Basel, Switzerland) according to the manufacturer's guide. After 48 h, cells transfected with pcDNA were harvested to detect the expression level of CASC2 by qRT-PCR.

### Cell proliferation assay

Cell proliferation was assayed using a cell proliferation kit (MTS) (Promega, Madison, WI) according to the manufacturer's protocol. Cells transfected were seeded into 96-well plates at a density of 3×103 cells/well in 100μl medium. Cell growth proliferation was assessed every 24 h according to the manufacturer's instructions. 20μl of MTS regent was added to each well and incubated at 37 °C for 2 h. Absorbance was recorded at 490 nm with Envision microplate reader (PerkinElmer). Experiments were performed in triplicate.

### Colony formation assay

Tumor cells transfected were seeded in 6-well plates at a density of 500 cells per well and incubated for 10-14 days for the colony formation assay. The cells were gently washed with PBS and then fixed with 4% paraformaldehyde and stained with 0.2% crystal violet (Sigma). Colonies were photographed and counted by Image J software (NIH, Bethesda, MD). The assays were performed in triplicates.

### Cell apoptosis and cycle flow cytometry assay

The bladder cancer cells transfected with pcDNA-CASC2 or empty vector were harvested for 48 h for cell apoptosis and cell cycle analysis. The cell apoptosis was determined by the Annexin V-FITC apoptosis kit (Sigma-Aldrich Chemical Company, St Louis MO, USA) according to the manufacturer's instructions. The Annexin V-FITC and PI fluorescence levels were measured by flow cytometry (BD Biosciences, FACS Calibur). The Annexin V-positive cells (both PI-negative and -positive) were defined as apoptotic cells. For the cell cycle analysis, cells were collected and fixed with 70 % pre-cooling anhydrous ethanol at −20 °C overnight and incubated with propidium iodide (PI) dye liquor (Sigma) in the presence of Rnase A (Promega) for 30 min at room temperature. The cell cycle distribution was analyzed by flow cytometry (FACSCalibur, BD Biosciences).

### Cleaved caspase-3 colorimetric assay

Briefly, bladder cancer cells T24 and 5637 were plated at 5 ×10^5^ cells/well in a 6-well plate for 24 h, then transfected with corresponding pcDNA-CASC2 or empty vector, respectively. At 48 h after transfection, cleaved caspase-3 activity were measured with the Caspase-3 Colorimetric Assay kit (R&D Systems, Minneapolis, USA) according to the manufacturer's recommendations. The assays were performed in triplicates.

### Migration and invasion assay

The 24-well transwell chamber with 8μm pores was purchased from BD (BD Biosciences, USA). Cells transfected (1 × 105 cells per well) were suspended in 100μl serum-free medium and then seeded to the upper chamber in the 24-well plate, and 600μl medium with 10% FBS was filled with the lower chamber. After incubating 24h at 37°C, the cells migrated into the lower membrane surface were fixed with methanol, stained with 0.5% crystal violet and then counted under a light microscope at ×100 magnification in five randomly selected fields across the center and the periphery of the membrane. For the cell invasion assay, the transwell membrane was pre-coated with 30 μl of Matrigel (1:3 mixed with PBS; BD Biosciences) and proceeded the same as described above. Experiments were performed in triplicate.

### Western blot analysis

Cells were lysed in a lysis buffer (10 mM KCl, 20 mM HEPES, 5 mM EDTA, 1% NP-40, 0.25% deoxycholate, pH 7.4) with protease and phosphatase inhibitors (1 mM Na3VO4, 10 mM NaF, 1 mM phenylmethanesulfonyl fluoride, 2 μg/ml aprotinin). Protein concentrations were measured by the BCA protein assay (Pierce, IL, USA). Equal amounts of the protein were denatured in sample buffer and then electrophoresed by 5-10% SDS-PAGE, transferred to the polyvinylidene fluoride (PVDF) membrane (no. IPFL00010; Merck Millipore, Darmstadt, Germany) under 100 V for 2 h and incubated with the following primary antibodies overnight at 4°C: anti-β-actin antibody (Cell Signaling Technology); Mouse monoclonal anti-E-cadherin (#14472, Cell Signaling Technology), rabbit polyclonal anti-β-catenin (ab6302, Abcam); mouse monoclonal anti-cycle D1 antibody(MA1-12296, Thermo Fisher Scientific); polyclonal anti-c-myc antibody (#9402, Cell Signaling Technology). The primary antibody incubation was followed by incubation with fluorescent Dye-tagged secondary antibodies that were 20,000-fold diluted in Odyssey blocking buffer (TBS) and an Odyssey Infrared Imaging System (LI-COR).

### Statistical analysis

All data were expressed as the means ± standard deviation (SD). Student's t test was used for differences comparisons between two independent groups. Data was analyzed using GraphPad Prism 5.0 (GraphPad Software, La Jolla, CA, USA). Statistically significant differences were defined as P <0.05.
